# New Insights into Hippo/YAP Signaling in Fibrotic Diseases

**DOI:** 10.3390/cells11132065

**Published:** 2022-06-29

**Authors:** Masum M. Mia, Manvendra K. Singh

**Affiliations:** 1 Cardiovascular and Metabolic Disorders Program, Duke-NUS Medical School, 8 College Road, Office 08-15, Singapore 169857, Singapore; masum.mia@duke-nus.edu.sg; 2 National Heart Research Institute Singapore, National Heart Centre Singapore, Singapore 169609, Singapore

**Keywords:** tissue fibrosis, fibroblast, myofibroblast, adverse remodeling, Hippo signaling, YAP, TAZ

## Abstract

Fibrosis results from defective wound healing processes often seen after chronic injury and/or inflammation in a range of organs. Progressive fibrotic events may lead to permanent organ damage/failure. The hallmark of fibrosis is the excessive accumulation of extracellular matrix (ECM), mostly produced by pathological myofibroblasts and myofibroblast-like cells. The Hippo signaling pathway is an evolutionarily conserved kinase cascade, which has been described well for its crucial role in cell proliferation, apoptosis, cell fate decisions, and stem cell self-renewal during development, homeostasis, and tissue regeneration. Recent investigations in clinical and pre-clinical models has shown that the Hippo signaling pathway is linked to the pathophysiology of fibrotic diseases in many organs including the lung, heart, liver, kidney, and skin. In this review, we have summarized recent evidences related to the contribution of the Hippo signaling pathway in the development of organ fibrosis. A better understanding of this pathway will guide us to dissect the pathophysiology of fibrotic disorders and develop effective tissue repair therapies.

## 1. Introduction

**Figure 1:**
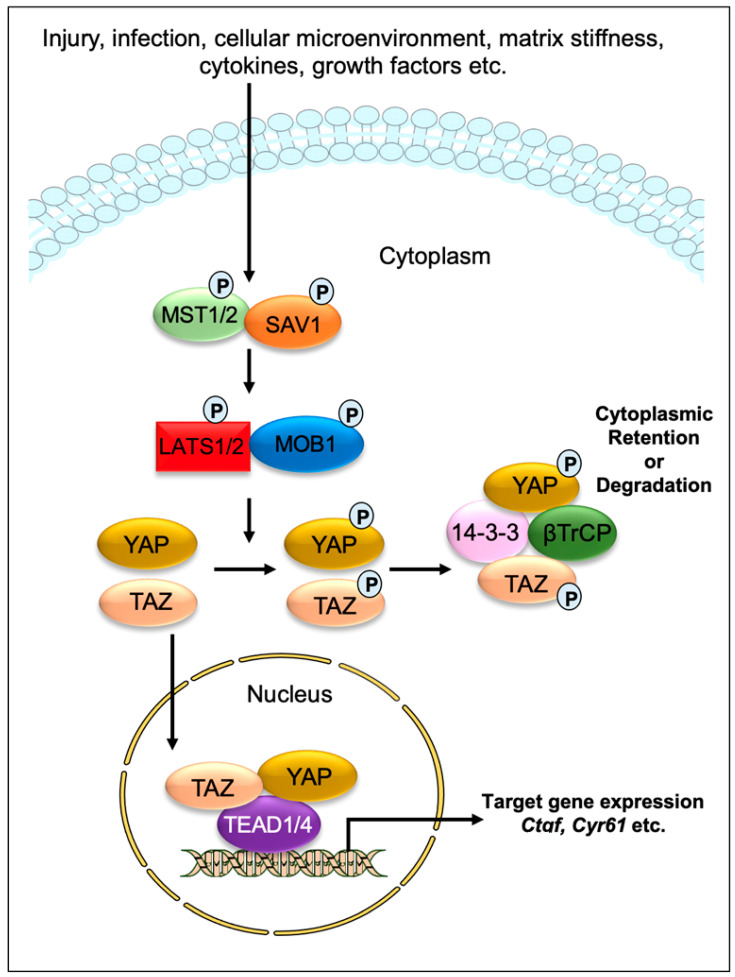
**Graphical demonstration of the Hippo signaling pathway**. Various physiological and pathological signals can induce the Hippo signaling pathway. In mammals, the core elements of the Hippo pathway mainly consist of serine/threonine kinases, transcriptional factors, and their cofactors. The transcriptional factors Yes-associated protein (YAP) and its coactivator PDZ-binding motif (TAZ/WWTR1) are the key effectors of the Hippo pathway. After physiological or pathological stimuli, sterile 20-like protein kinase (MST1/2) interacts, phosphorylates, and forms complexes with the adaptor protein Salvador 1 (SAV1), which phosphorylates large tumor suppressor (LATS1/2) and LATS1/2-interacting protein MOB kinase activator 1 (MOB1). The phosphorylated LATS1/2–MOB1 complex then phosphorylates YAP and TAZ, which promotes cytoplasmic retention or polyubiquitination and consequent degradation of YAP/TAZ by proteasomes during autophagy. However, dephosphorylation of upstream kinases leads to the nuclear translocation of YAP and TAZ, where they can interact with various transcription factors including TEA domain DNA-binding family members (TEAD1–4) and regulate the expression of Hippo pathway target genes such as *Ctgf* and *Cyr61*.

Impaired tissue repair processes due to the dysregulation of molecular and cellular events after organ injury lead to organ fibrosis, a common cause of organ failure after chronic injury and/or inflammation [1]. A hallmark of fibrosis is the excessive deposition of the collagen-rich extracellular matrix (ECM), especially due to an imbalance between collagen synthesis and remodeling. One of the key processes in fibrosis is the differentiation/transition of fibroblasts into myofibroblasts, the main fibrotic cellular phenotype, which produces a series of ECM components such as collagens, laminins, and fibronectins [2,3]. Numerous cell types, including epithelial and endothelial cells are also able to transdifferentiate into myofibroblastic phenotype (such as epithelial-mesenchymal transition and endothelial-mesenchymal transition) during organ fibrogenesis [4]. Recent studies have implicated the contribution of Hippo signaling pathway components in the fibrosis of various tissues, including the lung [5,6,7], liver [8,9,10,11,12,13,14], kidney [15,16,17], and heart [18,19,20]. The Hippo signaling pathway was first identified and illustrated in *Drosophila*. In mammals, the key components of the pathway are highly conserved, including several serine/threonine kinases and transcriptional factors, which are orthologs of *Drosophila* proteins. The transcriptional regulator Yes-associated protein (YAP) and its co-activator PDZ-binding motif (TAZ/WWTR1) are the main effectors of this pathway. In response to physiological or pathological stimuli, sterile 20-like protein kinase (MST1/2) forms complexes with the adaptor protein Salvador 1 (SAV1) that phosphorylates large tumor suppressors (LATS1/2) and LATS1/2-interacting protein MOB kinase activator 1 (MOB1). The phosphorylated LATS1/2–MOB1 complexes, in turn, phosphorylate YAP and TAZ, resulting in the cytoplasmic retention or polyubiquitination and subsequent degradation of YAP/TAZ by proteasomes during autophagy (Figure 1). In contrast, dephosphorylation of upstream kinase cascade drives the nuclear trafficking of YAP and TAZ, where they can interact with numerous transcription factors, including TEA domain DNA-binding family members (TEAD1–4), and regulate the expression of Hippo pathway target genes responsible for cell proliferation, survival, and differentiation [21,22,23].

The Hippo signaling pathway has been extensively studied for its role as an organ size controller that regulates cell proliferation, apoptosis, cell fate decisions, and stem cell self-renewal during development, homeostasis, regeneration, and cancer formation in numerous mammalian organs [21,22,24,25,26]. A growing number of studies have reported that the cascade components also play a critical role in fibrotic diseases. For example, the nuclear YAP/TAZ in resident cardiac fibroblasts obtained from preclinical myocardial infarction (MI) models [18,19,27], are sufficient to direct the transdifferentiation of cardiac fibroblasts into pathological myofibroblasts [27,28]. Similarly, YAP/TAZ are important regulators of pathological fibroblast activation in pulmonary fibrosis [5] and hepatic stellate cell activation in liver fibrosis [11,12,13,14]. Furthermore, activated YAP/TAZ in interstitial myofibroblasts promotes kidney fibrosis [16], while hyperactivated YAP/TAZ in non-fibroblast cells, such as macrophages [29,30], epithelial cells [31,32,33], and hepatocytes [8,10] contribute to the pathogenesis of fibrosis. Consistent with these findings showing the fibrotic effects of YAP/TAZ, their inactivation was found to be beneficial for preventing myofibroblast formation and fibrosis development [34]. Like YAP/TAZ, Hippo kinases are also known to contribute to a range of fibrotic diseases [20,35,36,37,38]. As such, treatment of mice with an MST1/2 blocker XMU-MP-1 inhibits pressure-induced cardiac hypertrophy and fibrosis (Table 1) [39]. Similarly, LATS1/2-mutant cardiac fibroblasts are less prone to develop into fibrotic phenotype after infarction-induced injury [35], while deletion of LATS1/2 stimulates YAP/TAZ activation in myofibroblasts and aggravates kidney fibrosis [40]. Similarly, genetic inactivation of SAV1 in renal tubule cells promotes renal interstitial fibrosis [36]. In fibrotic diseases, various profibrotic signals can induce the function of the Hippo effectors. For example, stiff substrates drive the nuclear localization of YAP and TAZ in fibroblasts and regulates their differentiation into myofibroblasts that promote profibrotic ECM synthesis; on the other hand, soft substrates prevent ECM synthesis. [5,15,17,41]. Therefore, targeting YAP/TAZ and their downstream signaling components could be an effective strategy for protecting various organs against fibrosis and related pathology.

## 2. Hippo Signaling Pathway in Cardiac Fibrosis

The fibrotic response is a crucial contributor to heart failure (HF), which occurs in many types of cardiac diseases, including cardiac ischemia, myocardial infarction (MI), and cardiac hypertrophy. Understanding the mechanisms underlying fibrotic cardiac remodeling after injury remains a critical barrier to developing effective treatments for HF patients. An increasing number of studies have revealed important roles for Hippo signaling components in the development and progression of cardiac fibrosis [18,19,20,23,27,28,29,35,37,39,42,43,44,45,46,47,48]. While YAP/TAZ activation is crucial for driving cardiac fibrosis, their suppression was beneficial for preventing angiotensin II (AngII) or MI-induced fibrosis [18,19,29,44].

**Figure 2:**
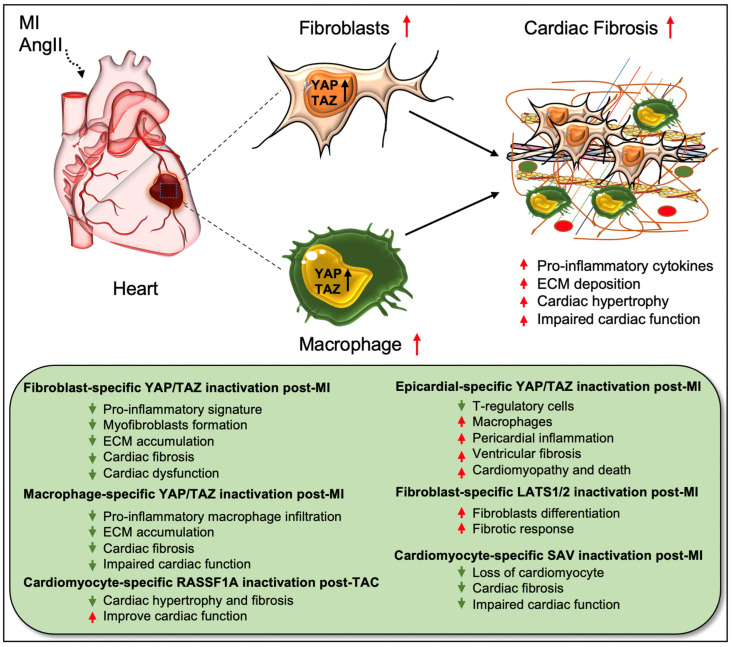
**Role of Hippo/YAP pathway in cardiac fibrosis and repair.** Activation of YAP/TAZ in fibroblasts and macrophages plays a crucial role in driving cardiac fibrosis after MI or AngII-induced injury. Fibroblast-specific loss of *Yap/Taz* or loss of only *Yap* improves cardiac outcome and reduces fibrotic response in infarcted hearts post-injury. Genetic ablation of *Yap/Taz* in fibroblasts blocks the proliferation of fibroblasts and their differentiation into pathologic myofibroblasts and disrupts macrophage-mediated inflammatory signature. In contrast, fibroblast-specific inactivation of upstream Hippo kinases *Lats1/2* aggravates fibrotic response post-MI. Like fibroblasts, conditional ablation of *Yap/Taz* in macrophages leads to impairment of macrophage-mediated inflammatory response and thereby reduces cardiac fibrosis, resulting in improved cardiac function and hypertrophy post-MI. Consistently, cardiomyocyte-specific inactivation of Hippo kinase *Sav* reduces cardiomyocyte apoptosis and cardiac fibrosis, which causes improved cardiac function post-MI. Likewise, cardiomyocyte-specific deletion of *Rassf1A* reduces TAC-induced cardiac hypertrophy and fibrosis with improved cardiac function. Epicardium-specific deficiency of *Yap/Taz* reduces the infiltration of T-regulatory cells (a subclass of adaptive immune cells) while increasing the recruitment of macrophages (a subclass of innate immune cells) in the myocardium, which promotes pericardial inflammation and ventricular fibrosis after MI-injury that subsequently leads to cardiomyopathy and death.

Cardiac fibroblasts are believed to be the major source of pathological ECM synthesis during cardiac remodeling. Elevated YAP and downregulated upstream Hippo kinase LATS1 were determined in the left ventricular tissue of HF patients, which was associated with cardiac fibroblast proliferation [42]. Recent studies also identified the activation of YAP and TAZ in resident cardiac fibroblasts from preclinical MI models (Figure 2) [18,19,27]. YAP and TAZ can directly induce the differentiation of fibroblasts into pathologic myofibroblasts [27,28]. Fibroblast-specific deletion of *Yap/Taz* using *Col1a2^Cre(ER)T^* mice [18] or deletion of only *Yap* using *Tcf21^MCM^*;*Yap^F^*^/*F*^ mice [19] showed reduced fibrotic and inflammatory responses in infarcted hearts. Similarly, *Yap/Taz* deletion resulted in an impaired profibrotic response in the fibroblasts from the infarcted heart. Fibroblast-specific *Yap/Taz* deficiency resulted in improved cardiac function in mice post-MI [18]. Loss of YAP also reduced myocardial fibrosis and cardiac dysfunction in response to chronic neuroendocrine stimulation by AngII. In vitro studies revealed that blocking of YAP/TAZ with siRNA or verteporfin abrogated TGF-β1-induced transition of fibroblasts into myofibroblasts and ECM (such as collagen and fibronectin) production [18]. In contrast, fibroblast-specific YAP overexpression using YAP^5SA^ mice promoted inflammatory response, fibrosis, and hypertrophy in the infarcted heart [18]. Consistently, Francisco J et al. [28] described that targeted overexpression of YAP in cardiac fibroblasts with adeno-associated virus construct AAV-hTCF21-FLAG-Yap(S127A)-augmented myocardial inflammation and fibrosis in mice, resulting in declined cardiac function. Mechanistically, ras homolog gene family member A (RhoA) regulates AngII-induced YAP activation, which mediates the transition of cardiac fibroblasts into fibrotic myofibroblasts [19]. Moreover, YAP interacts with MRTF-A (myocardin-related transcription factor A) to facilitate the formation of α-smooth muscle actin (α-SMA)-positive myofibroblasts and profibrotic gene expression [19]. YAP/TAZ also regulate interleukin-33 (IL-33) to promote cardiac myofibroblasts formation [18]. These studies suggest that YAP and TAZ are crucially involved in regulating the formation of cardiac myofibroblasts and fibrosis.

Xiao et al. [35] elucidated that fibroblast-specific inactivation of upstream Hippo kinases LATS1/2 using *Tcf21iCre*;*Lats1^fl^*^/*fl*^;*Lats2^fl^*^/*fl*^;*Rosa26^mTmG^* mice initiated a spontaneous fibrotic response in the adult heart, which was aggravated after MI injury. The study also showed that LATS1/2 inhibits the differentiation of resting cardiac fibroblasts to myofibroblast-like cells. At the molecular level, *Lats1/2* mutant-cardiac fibroblasts showed reduced fibrotic phenotype due to reduced YAP/TAZ expression after infarction [35]. Systemic loss of *Lats2* in mice alleviated myocardial fibrosis after transverse aortic constriction (TAC)-induced injury [37]. Likewise, Lats1/2 inactivation in cardiomyocytes was also found to be protective against fibrosis after MI. Using *αMHC^MerCreMer^*;*Sav^f^*^/*f*^ mice, Leach et al. [20] showed that *Sav* deletion in cardiomyocytes improved cardiac function and reduced cardiac fibrosis with an increased presence of left ventricular cardiomyocytes at 9 weeks post-MI. Del Re et al. [47] studied the crucial role of RASSF1A/MST1 pathway in TAC-induced cardiac fibrosis. RASSF1A is a physiological regulator of MST1 activation and the function of the RASSF1A/MST1 pathway is different between cardiomyocytes and fibroblasts. Overexpression of RASSF1A using *Rassf1A* transgenic mice increased MST1 phosphorylation, cardiomyocyte apoptosis as well as cardiac fibrosis upon pressure overload. Alternately, mutations in *Rassf1A* alleviated MST1 activation and improved cardiac function. Interestingly, systemic inactivation of *Rassf1A* also aggravated the fibrotic response following TAC in the heart. The authors observed that cardiomyocyte-specific ablation of *Rassf1A*, using *α-MHC^Cre/+^* mice, attenuated TAC-induced cardiomyocyte apoptosis, cardiac hypertrophy, and fibrosis, and led to improvement in cardiac function. Interestingly, silencing of *Rassf1A* in cardiac fibroblasts stimulated the TNFα-mediated proliferation of myofibroblasts and induced cardiac hypertrophy after pressure overload. These findings reveal cell-type-specific functions of RASSF1A after TAC-induced cardiac injury; inactivation of *Rassf1A* in cardiomyocytes has protective effects while its inactivation in cardiac fibroblasts is detrimental to the heart.

Recent research has also described the role of Hippo mediators in cells other than fibroblasts and cardiomyocytes. For instance, Ramjee et al. [46] demonstrated an epicardium-specific role of YAP/TAZ during myocardial recovery post-MI. Deletion of *Yap/Taz* in the epicardium, using *Wt1^CreERT2^*^/*+*^ mice, aggravated pericardial inflammation and ventricular fibrosis post-MI due to defective recruitment of regulatory T (Treg) cells (a subgroup of CD^4+^ T cells) into the injured myocardium, resulting in cardiomyopathy and death. As a causal link, the study team found that YAP/TAZ regulates IFN-γ activity in the activated epicardium, which is important for myocardial recovery after MI. Epicardium-specific inactivation of *Yap/Taz* caused reduced IFN-γ expression; the exogenous administration of IFN-γ post-MI by using a hydrogel system enhanced Treg cell recruitment into the injured myocardium and reduced fibrosis [46]. Similarly, another recent study revealed a macrophage-specific role for YAP/TAZ in myocardial fibrosis, where the proteins were shown to function as essential regulators of macrophage-mediated proinflammatory or reparative responses post-MI in the heart [29]. Conditional loss of *Yap/Taz*, using *LysM^Cre^* mice, decreased pro-inflammatory and improved reparative macrophage polarization in the infarcted heart, leading to reduced cardiac fibrosis as well as improved cardiac outcomes. In contrast, *Yap* overexpression, using a constitutively-active YAP mutant (YAP^5SA^) and *LysM^Cre^* mice, caused an increase in inflammatory macrophage polarization and impairment in reparative macrophage polarization. As a consequence, YAP overexpression leads to exacerbated cardiac fibrosis and defective remodeling in the infarcted heart. These findings indicate that YAP/TAZ regulate post-MI macrophage polarization, which influences cardiac fibrosis and repair processes and, thereby, cardiac function (Figure 2) [29]. Since epicardium-specific ablation of *Yap/Taz*-aggravated fibrosis and myeloid-specific inactivation reduced cardiac fibrosis and improved cardiac outcomes, targeted depletion of YAP/TAZ would be essential to prevent cardiac fibrosis post-MI.

Research over the years has revealed several components that have protective roles against cardiac fibrosis. For example, treatment of mice with MST1/2 blocker XMU-MP-1 prevented TAC-induced cardiomyocyte apoptosis, cardiac hypertrophy, and fibrosis [39]. Similarly, *Qishen* granules were shown to reduce the expression of fibrogenic proteins TGF-*β*1, SMAD3, and CTGF in rats with HF [43]. SKI, the cellular homolog of the avian Sloan-Kettering virus, negatively regulated the activation of primary rat cardiac fibroblasts by inducing proteasomal degradation of TAZ through interactions with LIM Domain-containing protein 1 (LIMD1) [27]. Furthermore, blocking of RhoA attenuated AngII-induced YAP/TAZ transcriptional activity [19] and suppression of YAP/TAZ signaling in mice by lovastatin alleviated AngII-induced fibrosis, both in vitro and in vivo (Table 1) [44]. The gaseous signaling molecule sulfur dioxide (SO2) efficiently prevented myocardial fibrosis by downregulating Hippo kinase MST1/2 in diabetic rats [45].

## 3. Hippo Signaling Pathway in Pulmonary Fibrosis

Pulmonary fibrosis (PF) is an interstitial lung disease associated with a high mortality rate, and is characterized by aberrant inflammatory response, progressive scarring due to excessive accumulation of pathologic fibroblasts and extracellular matrix (ECM) between the alveoli or air sacs, and destruction of lung parenchyma. This results in deteriorated oxygen exchange function and breathlessness that over time can lead to respiratory failure and death. The decline in respiratory function after an injury is associated with the dysregulation or loss of Alveolar epithelial type I (AT1) and type II (AT2) cells [49]. AT2 cells act as stem/progenitor cells that are capable of self-renewal and differentiation into AT1 cells during both homeostatic state and reparative phase post-injury [50]. Dysregulation of AT2 to AT1 differentiation after injury has been reported in animal models and patients with PF [51]. Several factors are known to initiate PF including aging, genetic disorders, autoimmune diseases, radiation, environmental factors, and most recently SARS-CoV-2 infection [52,53,54]. Idiopathic pulmonary fibrosis, a condition in which the cause of PF is unknown, occurs mostly in older adults, with a median survival of 2–5 years post-diagnosis [55]. Currently available treatments, including use of anti-fibrotic and anti-inflammatory drugs pirfenidone and nintedanib, have shown to reduce deterioration in lung function in patients with idiopathic PF. However, deaths related to idiopathic PF still persist [52,56,57]. Recent findings reported an activation of Hippo effectors YAP/TAZ in alveolar epithelial cells (AECs) and fibroblasts that was associated with either lung regeneration or adverse remodeling after injury, including IPF, [5,38,58,59,60,61,62]. This suggests that the YAP/TAZ pathway might be an interesting target for designing anti-fibrotic therapy.

**Figure 3:**
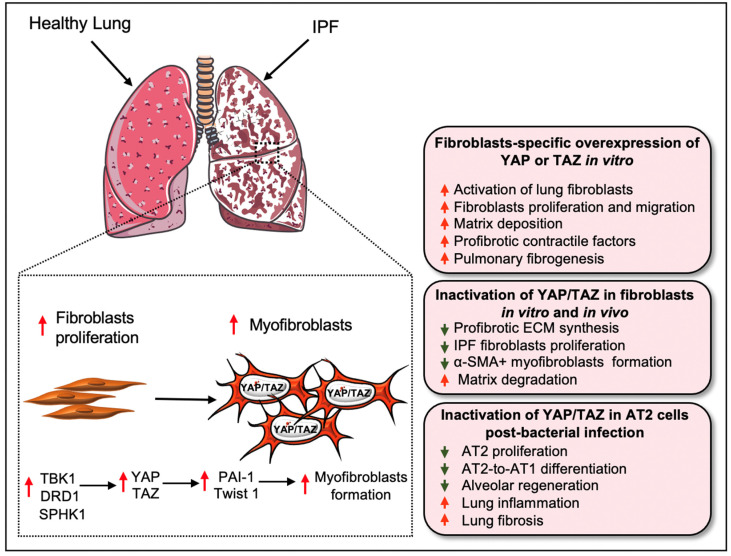
**Role of YAP/TAZ in pulmonary fibrosis and regeneration.** In the healthy lung, YAP/TAZ (i.e., YAP/TAZ activity) is almost absent in the nucleus. However, aberrant expression of YAP/TAZ was detected in the lungs of IPF patients. Matrix stiffness or profibrotic stimuli (such as TGF-β1) directs the nuclear localization of YAP/TAZ on cultured fibroblasts and activation of YAP/TAZ augments the proliferation and differentiation of lung fibroblasts into myofibroblasts, which increases ECM synthesis. At the molecular level, several biological components including TBK1, DRD1, and SPHK1 promote YAP/TAZ activity in fibroblasts for its differentiation into pathogenic myofibroblasts in a profibrotic environment. The profibrotic effects of YAP and TAZ could be further regulated through transcriptional interactions with PAI-1 and Twist1. Inactivation of *Yap/Taz* in fibroblasts reduces their proliferation and myofibroblasts formation. Silencing of *Yap/Taz* also interrupts ECM synthesis by promoting matrix degradation process. On the contrary, overexpression of YAP or TAZ increases the fibrogenic potential of the fibroblasts to induce excessive matrix deposition and progression of pulmonary fibrosis. However, YAP/TAZ activation in AT2 serves a protective role during bacterial infection in the lungs. The nuclear presence of YAP/TAZ in AT2 cells regulates the proliferation and differentiation of AT2 into AT1, an essential process in lung regeneration, after *Streptococcus pneumoniae* strain T4 (SpT4)-induced lung injury. In contrast, AT2-specific *Yap/Taz* deletion promotes lung inflammation and fibrosis, decreases AT2 proliferation and AT2-to-AT1 differentiation, and thus abrogates alveolar regeneration in the infected lung.

Recent findings by Liu and colleagues demonstrated that YAP and TAZ are essential regulators of pathological fibroblast activation in PF (Figure 3) [5]. YAP and TAZ were abundantly expressed in fibroblasts derived from the region of fibrosis in IPF patients. Furthermore, the TAZ showed a pronounced nuclear localization in the IPF fibroblasts, while, in the healthy lung, YAP and TAZ were largely absent at the nucleus. The study team demonstrates that matrix stiffness directs the nuclear localization of YAP and TAZ on cultured fibroblasts and siRNA-mediated knockdown of the two proteins significantly reduced profibrotic ECM synthesis and IPF fibroblasts proliferation. They further showed that activation of YAP/TAZ using the YAP5SA or TAZ4SA construct augmented fibroblasts proliferation and *COL1A1* transcripts synthesis on cultured NIH3T3 cells. The injection of YAP5SA- or TAZ4SA-containing cells into mice using the adoptive cell transfer model promoted matrix deposition in the lung, suggesting that YAP or TAZ is sufficient to increase the fibrogenic potential of the fibroblasts [5]. Mechanistically, YAP/TAZ regulates matrix-stiffness-dependent fibroblast activation through its target gene *SERPINE1*, which encodes plasminogen activator inhibitor 1 (PAI-1). Knockdown of YAP/TAZ expression was sufficient to interrupt TGF-β-induced expression of PAI-1. In this study, the profibrotic effects of YAP and TAZ were partially regulated through PAI-1, which could be regulated by matrix stiffness independent of transforming growth factor-β (TGFβ) signaling [5]. YAP/TAZ are an important regulator of fibroblast contractile function during alveologenesis and PF. Combined knockdown of YAP/TAZ in human lung fibroblasts from patients with idiopathic pulmonary fibrosis reduced the expression of three profibrotic contractile genes, *Acta2*, *Cnn1*, and *Tagln*. Similarly, knockdown of YAP/TAZ was sufficient to interrupt the TGF-β-induced lung fibroblast activation [6].

Another study reported that fibroblasts-specific selective targeting of YAP/TAZ is necessary to reverse experimental lung fibrosis since non-cell-specific targeting of YAP/TAZ amplified lung fibrosis [7]. Selective inhibition of YAP/TAZ through the agonism of Gα_s_-coupled dopamine receptor D1 (DRD1), a G protein-coupled receptor (GPCR), in the IPF fibroblasts reversed the formation of profibrotic α-SMA-positive myofibroblasts and augmented matrix degradation in a TGFβ-rich profibrotic environment [7]. GPCRs are known to regulate YAP activity in profibrotic conditions [63,64] and GPCR ligands are also important co-factors of nuclear YAP. For example, the GPCR ligand S1P was found to be elevated in IPF [63] and a positive correlation between nuclear YAP and GPCR ligands such as LPA, S1P, thrombin were detected in fibroblasts during TGFβ1-induced fibrotic responses (Table 1) [64], suggesting GPCRs-YAP axis is crucial for fibroblast activation.

Hippo/YAP functions through several other signaling pathways during IPF [65]. Huang et al. showed that the genetic deletion of sphingosine kinase 1 (*Sphk1*) in fibroblasts and AECs reduced YAP1 expression and protected the mice from bleomycin-induced lung fibrosis. Similarly, genetic deletion or the inhibition of SPHK1 activity by PF543 (a SPHK1 specific inhibitor) reduced TGF-β-mediated expression of YAP1 and profibrotic components fibronectin and α-SMA in lung fibroblasts from wild type mice. Furthermore, the blocking of Sphingosine-1-phosphate (S1p) receptors using antibodies dampened TGFβ-mediated YAP activation. Overall, this study provided insights into how SPHK1/S1P signaling regulates the profibrotic function of YAP in lung fibroblasts [65]. Recently, Tank binding protein kinase-1 (TBK1) was also identified as an important regulator of TGF-β-mediated lung fibroblast activation through its functions in maintaining YAP/TAZ stability [66]. Chemical or siRNA-mediated inhibition of TBK1 led to a decrease in the nuclear levels of YAP/TAZ, α-SMA stress fibers, and accumulation of ECM components such as collagen I and fibronectin in TGF-β-stimulated IPF fibroblasts.

Another study on primary human lung fibroblasts by Santos et al. [67] identified HMG-CoA (hydroxymethylglutaryl-coenzyme A) reductase inhibitors (commonly referred to as “statins”) for YAP targeting through high-throughput small-molecule screening. Statins impede YAP activation independently of Hippo signaling and inhibit profibrotic fibroblast differentiation. This study found that simvastatin modulates YAP localization in mouse lung fibroblasts in vivo and reduces established fibrosis in bleomycin-challenged mouse IPF (Table 1) [67]. Furthermore, Chen et al. [68] described that YAP1 overexpression augmented the expression of collagen, fibronectin, and Hippo/YAP target CTGF in fibroblasts. Similarly, YAP1 enhanced the proliferation, migration, and transition of cultured lung fibroblasts into myofibroblasts. The study team demonstrated that YAP1 augments fibrotic events through the transcriptional activation of Twist1 by interacting with its partner TEAD [68]. Knockdown of YAP1 reduced ECM deposition and attenuated lung fibrosis. Furthermore, they identified that miR-15, a microRNA whose levels is reduced in IPF patients, acts as a crucial regulator of the YAP1/Twist pathway and has the potential to inhibit fibrogenesis by decreasing YAP expression in IPF [68]. A recent study demonstrated the beneficial effect of melatonin, a neurohormone, in preventing PF [69]. This study demonstrated that melatonin reduces interstitial lung fibrosis during bleomycin-induced IPF (Table 1), and attenuates TGF-β1-induced myofibroblast formation and collagen and fibronectin expression in lung fibroblasts. Furthermore, melatonin also interfered with fibroblast migration and prevented fibrogenesis by interrupting the YAP1 translocation from the cytoplasm to the nucleus. However, inhibition of melatonin receptors using luzindole reduced the anti-fibrotic effects induced by melatonin [69]. Icariin, another bioactive compound, was found to be effective in the inhibiting YAP functions, leading to the attenuation of bleomycin-induced PF in the rat (Table 1) [70]. Like YAP, TAZ activation was also upregulated in IPF fibroblasts [71]. Noguchi et al. found that increased TAZ expression in human lung fibroblasts was associated with myofibroblast formation, as described by the increased expression of α-SMA and CTGF [71].

During IPF, insufficient and defective epithelial regeneration drives the pathological remodeling of the injured lung [52,72]. YAP and TAZ are critically associated with epithelial regeneration after injury [38,59,61,62]. Gokey et al. [38] observed an increased nuclear localization of YAP, but a decreased MST1/2 expression in IPF epithelial cells. During IPF pathogenesis, YAP interacts with the mTOR/PI3K/AKT signaling axis to regulate proliferation, migration, differentiation, and polarity of lung epithelial cells [38]. Consistent with this, nuclear YAP/TAZ in AT2 cells was shown to regulate the proliferation and differentiation of AT2 into AT1 following *Streptococcus pneumoniae* strain T4 (SpT4)-induced lung injury [59]. The conditional deletion of *Yap*/*Taz* and lineage tracing of SPC-positive AT2 cells, using *SPC^CreERT2^*, *Yapf^l^*^/*fl*^, *Tazf^l^*^/*fl*^, and *Rosa26^mTmG^* mice, revealed an inflammatory signature with severe fibrotic lesions in the lung after SpT4 infection. *Yap/Taz* deletion also resulted in a decrease in AT2 proliferation and AT2-to-AT1 differentiation, thus impairing alveolar regeneration in the lungs exposed to SpT4 infection [59]. In addition, non-cell-specific targeting of YAP/TAZ with siRNA aggravated bleomycin-induced PF in mice by preventing alveolar regeneration [7]. These findings indicate that the YAP/TAZ pathway regulates the proliferation of both fibroblasts and alveolar epithelial cells during lung injury and alveolar regeneration (Figure 3). Although the role of YAP/TAZ has been studied in IPF fibroblasts and epithelial cells, further research will be needed to understand their role in inflammatory cells, including macrophages, and related inflammatory responses at different stages of IPF.

## 4. Hippo Signaling Pathway in Liver Fibrosis

During liver injury, activation of hepatocytes and non-hepatocytes such as hepatic stellate cells (HSCs), resident macrophages, and reactive-appearing duct-like cells (RDC) play a crucial role in the pathology of fibrotic nonalcoholic steatohepatitis (NASH), a pathologic condition described by the deposition of fat in the liver along with inflammation, hepatocyte death, and fibrosis. NASH results from an obesity-related liver disease, namely nonalcoholic fatty liver disease (NAFLD), occurring as a result of steatosis and related inflammation in the liver [73,74,75,76]. The role of Hippo pathway members has been extensively studied in liver injury, regeneration, and cancer development [77,78,79,80,81,82,83,84,85,86,87,88]. Recently, an essential role for YAP and/or TAZ in liver diseases, including NASH fibrosis, has been reported both in patients and pre-clinical mouse models [8,9,10,89].

**Figure 4:**
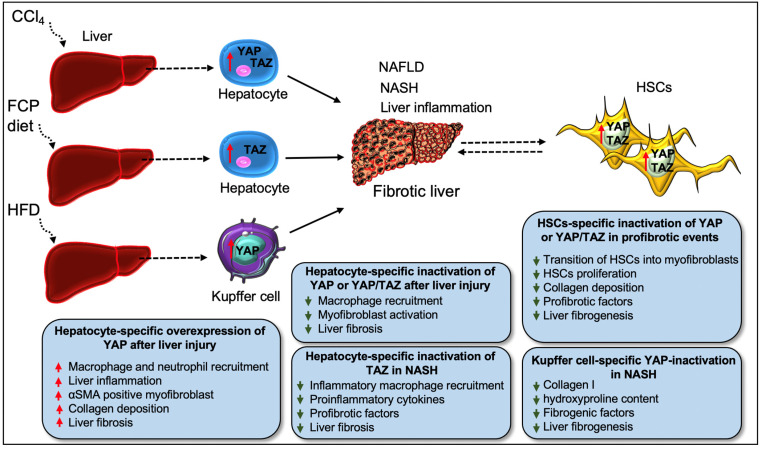
**Roleof YAP/TAZ in liver fibrosis.** Diet (HFD, FCP)- or chemical (CCl_4_)-induced liver injury leads to hepatic inflammation as well as NAFLD and NASH-related fibrosis, with YAP/TAZ playing a crucial role in the pathology during disease progression. Chronic liver injury (as initiated by the administration of CCl_4_) leads to YAP/TAZ hyperactivity in hepatocytes throughout the liver parenchyma, a process linked to the development of liver fibrosis. TAZ activation in hepatocytes alone can induce liver inflammation and NASH fibrosis. *Taz* deficiency in liver hepatocytes decreases the infiltration of inflammatory macrophages and the expression of profibrotic factors, which results in a decline in the inflammatory process and the development of fibrotic NASH in FCP-diet-fed (NASH diet) mice, a model comparable to human NASH. Similarly, deficiency of YAP or both YAP/TAZ in hepatocytes impairs macrophage infiltration and fibrogenic events such as myofibroblast formation and fibrosis after liver injury. However, YAP overexpression in hepatocytes triggers inflammation-mediated liver fibrosis, as described by the augmented presence of inflammatory cells (such as macrophages and neutrophils), αSMA-positive myofibroblasts, and collagen deposition in the injured liver. YAP/TAZ also regulates the transition of HSCs (the main source of pathologic myofibroblasts) in liver fibrosis. Pharmacological blocking of YAP/TAZ in HSCs is beneficial for preventing the activation, proliferation, and for stimulating profibrotic response (such as collagen accumulation) and liver fibrogenesis in these pathologic cells. In addition to hepatocytes and HSCs, YAP activation in Kupffer cells also contributes to hepatic inflammation during NASH progression. Myeloid-specific *Yap1* knockout mice show decreased liver fibrosis after CCl_4_ injury, as described by the lesser amount of α-SMA, collagen I, and hydroxyproline content due to macrophage-specific *Yap1* deletion. Likewise, myeloid-specific inactivation of YAP inhibits liver fibrogenesis in a diet-induced NASH murine model.

The role of hepatocytes is critical for the metabolic and detoxifying functions of the liver. As hepatocytes respond to any foreign small substances or infectious materials that pass through the intestine, they are exposed to a range of biochemical stresses, resulting in their death. This occurs through a process that stimulates inflammatory factors responsible for the initiation of liver inflammation and myofibroblast activation, which promotes liver fibrosis, and eventually, cirrhosis [74,90,91,92]. Recently, elevated activity of YAP/TAZ in hepatocytes has been described throughout the liver parenchyma during CCl4-induced chronic liver injury, a common model to study chronic liver injury-induced fibrosis [8]. Elevated YAP activity, correlating with the severity of hepatocyte injury, was found in hepatocytes, perivascular cells, and bile duct cells in the monkey model of NAFLD (Figure 4) [93]. An increased YAP/TAZ expression was also observed in liver hepatocytes exhibiting alcoholic liver disease or metabolic features of NASH [8]. Mooring et al. [8] studied the involvement of YAP/TAZ in hepatocytes that directly regulate macrophage-mediated liver inflammation and fibrosis after injury. Tetracycline-induced overexpression of YAP S127A in mice hepatocytes resulted in increased collagen deposition 2 weeks after intravenous delivery of a hepatocyte-specific Cre-recombinase (AAV-Cre). Consequently, more than 70% of the liver parenchyma showed exaggerated collagen deposition at 6 weeks after YAP-Tg induction, and YAP expression was found to be associated with αSMA-positive myofibroblast formation. The findings from this study demonstrated that YAP is required to trigger inflammation-mediated liver fibrosis, as determined by the augmented presence of immune cells such as neutrophils and macrophages in the liver at 1 week after YAP-Tg induction. To investigate the contribution of inflammation in fibrosis development, the study team depleted monocytes/macrophages in YAP-Tg mice through the administration of clodronate liposomes. The depletion of monocytes/macrophages resulted in decreased expression of ECM and its remodeling genes such as *Col1a1* and *Timp1*, with a minimal presence of αSMA-positive myofibroblasts. Loss of *Yap* or *Yap/Taz* in hepatocytes caused reduced macrophage infiltration and myofibroblast activation, which affects the development of liver fibrosis. Furthermore, the study found that CYR61 is an essential macrophage chemoattractant of the Hippo pathway. Overexpression of YAP-S127A promoted the expression of CYR61, also known as CCN1, in mice hepatocytes challenged with CCl_4_. Gain- and loss-of-function studies of CYR61 in vitro and in vivo revealed that YAP/TAZ-induced activation of CYR61 recruits the macrophages to promote liver fibrosis [8].

Another study by Wang et al. described an elevated level of TAZ in hepatocytes from patients with NASH fibrosis [10]. To understand the role of TAZ in NASH fibrosis, the study team silenced the *Taz* in liver hepatocytes using the adenovirus construct AAV8-H1-shTaz. Knockout of *Taz* in liver hepatocytes decreased the recruitment of inflammatory macrophages, pro-inflammatory components, and attenuated liver fibrosis in FCP-fed diet (fructose, palmitate, cholesterol-rich diet) mice, a model that mimics human NASH. They further investigated the contribution of TAZ in NASH using another model of mice, hyperphagic melanocortin-4 receptor knockout mice (*Mc4r*^−/−^) fed with an FPC diet. Compared to wild-type mice, *Mc4r*^−/−^ mice fed with FPC diet developed increased liver fibrosis. However, *Taz* silencing attenuated liver inflammation and fibrosis in FCP-fed *Mc4r*^−/−^ mice due to reduced macrophage recruitment and expression of profibrotic factors including *Tgfβ1*, and *Col1a1*. They confirmed that the treatment of mice with the AAV8-H1-shTaz construct reversed inflammation and fibrotic NASH development. An elevated TAZ expression in hepatocytes was also associated with increased expression of the TAZ target gene Indian hedgehog (*Ihh*) during NASH progression. Using several in vitro and in vivo studies, the authors found that TAZ-mediated activation of *Ihh* in hepatocytes stimulates HSCs-mediated fibrosis. Interestingly, Ihh restoration in the hepatocytes of TAZ-silenced mice re-initiated fibrotic-NASH progression, while direct silencing of *Ihh* in hepatocytes inhibited NASH development [10]. In a separate study, Wang et al. [9] found a correlation between higher levels of liver cholesterol and elevated TAZ levels and increased fibrosis in NASH mice. Mechanistically, cholesterol stabilizes TAZ by activating RhoA, a member of the GTPase. Silencing of *RhoA* in hepatocytes (using AAV8-H1-shRhoa vector) reduced the expression of TAZ and YAP while augmenting LATS1/2 activity. *RhoA* silencing in hepatocytes reduced the recruitment of inflammatory cells and attenuated liver fibrosis. Interestingly, *Taz* silencing in hepatocytes (using AAV8-H1-shTaz construct) did not affect YAP protein expression [9], suggesting that TAZ can promote NASH without YAP activity (Figure 4).

In the liver, HSCs are the principal source of pathologic myofibroblasts; HSC activation during chronic liver injury leads to enormous deposition of ECM which drives fibrosis, loss of function, and ultimately cirrhosis [74]. YAP/TAZ signaling is a crucial regulator of HSC activation in liver fibrosis [11,12,13,14]. YAP was found to be localized in the nucleus in HSCs derived from fibrotic livers in patient and mice models, while YAP nuclear localization was not observed in healthy livers [94]. The pharmacological blockade of YAP with verteporfin prevented HSC activation and collagen formation in cultured mouse HSCs and reduced fibrogenesis in mice exposed to chronic CCl_4_ treatment (Table 1) [13,94,95]. Martin et al. [95] identified that YAP is a core mediator of pro-fibrotic integrin signaling. Integrin β1 is required for the activation of pro-fibrotic HSCs and ablation of integrin β1 in HSCs impedes the induction of fibrotic markers and reduces YAP expression and its nuclear localization [95]. Kuo et al. [12] found that Hedgehog signaling promotes the differentiation of HSCs into myofibroblasts by activating YAP. They showed increased glutaminolysis in the HSCs of livers from patients and mice with acute or chronic fibrosis, and the inhibition of glutaminase abrogated the presence of myofibroblasts and fibrosis progression. Blockade of Hedgehog and YAP with their inhibitors impaired glutaminolysis and suppressed myofibroblastic features of HSCs [12]. These findings suggest that YAP is required for HSC activation and inhibition of YAP is necessary for the treatment of liver fibrosis. YAP/TAZ are also critical regulators of HSC proliferation and activation after ischemia-reperfusion injury (IRI) [96]. Treatment of mice with the YAP/TAZ inhibitor verteporfin significantly reduced HSC proliferation in post-ischemic liver. In contrast, a separate study demonstrated that activation of YAP in mice pre-treated with a YAP activator, 1-oleoyl lysophosphatidic acid (LPA), protected the liver from IRI, evident by reduced IRI-induced liver damage and liver fibrogenesis [97], while inhibition of YAP with verteporfin aggravated liver IRI. Mechanistically, YAP activation suppressed the infiltration of immune cells, including macrophages and neutrophils, and reduced the activation of HSCs to abolish IR-mediated liver fibrosis [97]. The beneficial function of LPA-induced YAP is interesting in liver IRI, and future studies are needed to understand the underlying molecular mechanisms. Another study on HSCs described that YAP/TAZ promotes the differentiation of HSCs into myofibroblasts in response to in situ stiffening which mimics the dynamics of mechanotransduction in liver fibrosis [41]. When HSCs were cultured on soft substrates, they retained their round morphology, whereas on stiff substrates they assumed a myofibroblast-like morphology. These findings suggest that YAP and TAZ are mechano-sensitive to substrate stiffness. HSCs on all stiffened substrates revealed an elevated level of YAP/TAZ nuclear intensity, α-SMA stress fiber formation, and fibrogenic *Acta2* and *Col1a1* expression compared to HSCs on soft gels. Thus, targeting YAP/TAZ signaling in HSCs might be a vital part of therapeutic intervention in liver fibrosis. For instance, pharmacological inhibition of acid ceramidase (aCDase) through tricyclic antidepressants in human HSCs or genetic knockout of *aCDase* in mouse HSCs reduced HSC activation, YAP/TAZ activity, and fibrosis (Table 1) [14]. Similarly, a dietary component, namely omega-3 polyunsaturated fatty acids (ω-3 PUFAs), has been reported to prevent the proliferation and activation of HSCs by promoting YAP/TAZ degradation [98]. Along these lines, dihydrexidine, a DRD1 agonist which blocks YAP/TAZ, was effective in reversing the HSC activation of bile duct ligation-induced hepatic fibrosis (Table 1) [7]. Similarly, Morin, a dietary flavonoid, was reported to block HSC activation by activating MST1 and LATS1 expression while decreasing the expression of YAP/TAZ [99]. Morin also alleviated liver fibrosis in diethylnitrosamine-induced rats (Table 1).

Recent evidence indicates that YAP plays a fibrogenic role in macrophages. Song et al. [30] studied a Kupffer cell-specific role for YAP during NASH progression. Myeloid-specific genetic loss of *Yap* or pharmacological inhibition of YAP with verteporfin reduced hepatic inflammation in mice fed with HFD (high-fat diet), compared to wild-type animals [30]. Elevated YAP levels were found in macrophages from the liver samples of human cirrhotic patients and CCl4-induced fibrotic mice models [100]. Myeloid-specific *Yap1* knockout mice using *LysM^Cre^* mice attenuated liver fibrosis after chronic CCl_4_ injury as the amount of α-SMA, collagen I, and the hydroxyproline content were impaired in *Yap1* knockout mice. Similarly, myeloid-specific loss of YAP impedes liver fibrogenesis in a diet-induced NASH murine model. This study further showed that ablation of *Yap1* activates type I interferon response, which reduces the expression of fibrogenic factors CTGF and vascular cell adhesion molecule-1 (VCAM1) and thereby decreases liver fibrogenesis. Treatment of the macrophages with a DRD2 antagonist (Fluphenazine dihydrochloride) suppressed YAP activity and induced type I interferon signaling (Table 1). Consistent with this, antagonism of DRD2 or myeloid-specific genetic ablation of *Drd2* reduced CCl4-induced liver fibrosis. Similarly, in NASH and bile duct ligation mouse models, DRD2 deletion in macrophages impaired liver fibrosis [100]. These observations suggest the targeting of DRD2/YAP-axis could be a potential approach to halt liver fibrogenesis. However, the role of DRD2 antagonists against TAZ activity remains to be studied further. It would be promising to understand whether DRD2 antagonists affects HSC activation in liver fibrogenesis.

The role of YAP has been also studied in other non-hepatocytes such as liver sinusoidal endothelial cells (LSECs) [101] and reactive-appearing duct-like cells (RDCs) [89]. Machado et al. showed that YAP expression in the liver of patients with NAFLD was associated with lobular inflammation and fibrosis in liver injury (Figure 4) [89]. They observed that YAP-expressing cells were mostly RDCs and the nuclear localization of YAP in RDCs was correlated with hepatocyte death during NASH. Increased expression of YAP in RDCs has also been associated with the mRNA levels of pro-fibrogenic factors (*Tgfβ1*, *Ctgf*, and phospho-SMAD2) and proinflammatory factors (F4/80 and TNF-α) in mouse NASH [89]. A study by Zhang et al. [101] found that YAP stabilized the levels of hypoxia-inducible factor 1α in LSECs and promoted angiogenesis in CCl_4_-induced liver fibrosis. These findings suggest that YAP function in various liver cells contributes to the development and progression of NASH fibrosis. However, it would be interesting to investigate whether TAZ functions in RDCs or LSECs also modulate hepatocyte injury and fibrosis during NASH/NAFLD.

## 5. Hippo Signaling Pathway in Renal Fibrosis

**Figure 5:**
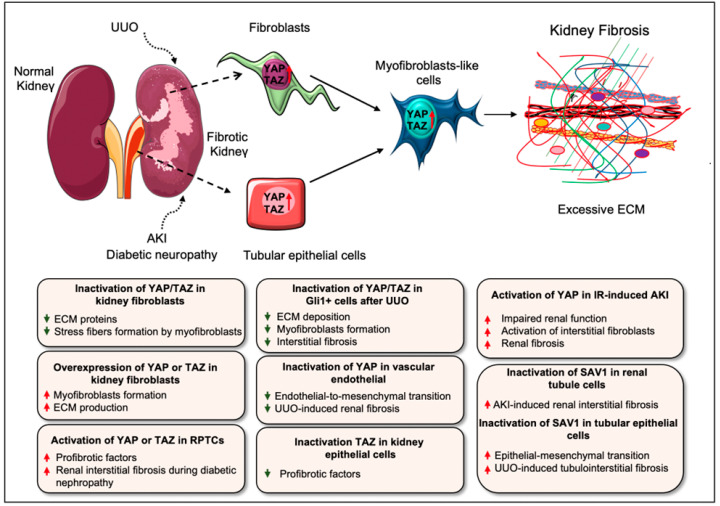
**Role of YAP/TAZ in kidney fibrosis.** Activation of YAP/TAZ has been reported in several types of kidney injury associated with fibrogenesis including UUO, AKI, and diabetic nephropathy in mice. Elevated levels of YAP/TAZ are found in renal fibroblasts/myofibroblasts during interstitial fibrosis after UUO-injury. Similarly, YAP activation in the post-acute phase of IR-induced AKI impairs renal function and aggravates fibrosis. YAP activation in renal proximal tubule epithelial cells also drives renal interstitial fibrogenesis during diabetic neuropathy. Consistently, UUO-induced renal injury and diabetic nephropathy in mice promote the nuclear accumulation of TAZ in the tubulointerstitium, which results in the progression of kidney fibrosis. In primary kidney fibroblasts, YAP/TAZ silencing impairs profibrotic stimuli–induced stress fiber formation and ECM synthesis while constitutive activation of YAP augments myofibroblasts formation and ECM production. Along these lines, constitutive activation of TAZ leads to the differentiation of fibroblasts into myofibroblasts. Likewise, overexpression of YAP or TAZ in renal tubular epithelial cells promotes the expression of fibrogenic factors, which stimulates the activation of interstitial fibroblasts. Selective targeting or inactivation of YAP/TAZ has protective role during UUO-induced renal fibrosis, fibroblast activation, and endothelial-to-mesenchymal transition (a myofibroblast-like cells) in vitro and in vivo. Like YAP/TAZ, Hippo kinase SAV1 also plays an essential role in renal interstitial fibrosis. *Sav1* inactivation in renal tubule cells augments renal interstitial fibrosis after AKI-induced injury. As such, tubular epithelial cell-specific deletion of *Sav1* increases myofibroblastic EMT (epithelial-mesenchymal transition)-like cells and aggravates tubulointerstitial fibrosis after UUO.

Renal fibrosis resulting from various renal injuries leads to progressive kidney damage and chronic kidney disease (CKD). Recent investigations reported that Hippo pathway components are crucial drivers of renal fibrosis [15,16,31,33,102,103]. Elevated YAP levels were reported in renal fibroblasts during interstitial fibrosis induced by unilateral ureteral obstruction (UUO) in mice [15]. Knockout of YAP/TAZ with Adenovirus-Cre impaired the TGF-*β*1–induced expression of ECM proteins (fibronectin and collagen I) and myofibroblast markers (*α*-SMA and SM22) in primary kidney fibroblasts, whereas constitutive activation of Yap (YAP5SA) augmented myofibroblasts formation and ECM production independent of TGF-*β*1 stimulation (Figure 5) [15]. The authors also showed that Gli1^+^ cell-specific genetic deletion of *Yap/Taz* using *Gli1^ERCre^;Yap^f^*^/*f*^*;Taz^f^*^/*f*^ mice attenuated UUO-induced ECM deposition, myofibroblast formation, and interstitial fibrosis. Similarly, the YAP inhibitor, verteporfin, reduced UUO-induced interstitial fibrosis in mice [15]. Liang et al. further showed that ECM stiffness could activate YAP to induce fibroblast activation [15]. Along these lines, Szeto et al. [17] described that ECM stiffness enhances TGF-*β*-induced profibrotic Smad signaling by regulating the localization of Smad2/3, a process mediated by the mechanoregulators YAP and TAZ. However, verteporfin treatment inhibited TGF-*β*-induced Smad activation and thereby impaired UUO-induced renal fibrosis and fibroblasts activation in cultured cells [17]. Myofibroblast-specific ablation of YAP/TAZ attenuated kidney fibrosis, while myofibroblast-specific deletion of LATS1 and LATS2 promoted YAP/TAZ activation in myofibroblasts and aggravated fibrosis [40].

Gui et al. [16] described that YAP/TAZ and mammalian target of rapamycin complex 2 (mTORC2) are activated in the interstitial myofibroblasts after UUO. Consistently, TGFβ1-mediated induction of YAP/TAZ and mTORC2 is required for renal fibroblast activation in vitro. Inhibiting either mTORC2 or YAP/TAZ signaling abrogated TGFβ1-induced fibroblast activation. Interestingly, blocking of mTORC2 with PP242, an mTOR kinase inhibitor, halted the expression of YAP/TAZ and their target genes *CTGF* and ankyrin repeat domain 1 (*ANKRD1*) in cultured fibroblasts and UUO-induced nephropathy (Table 1). Moreover, the overexpression of constitutively active form of Taz (Taz-S89A) was able to restore fibroblast activation blocked by PP242. These observations suggest that the targeting of mTORC2 could suppress YAP/TAZ pathway during fibroblast activation in kidney fibrosis [16]. Selective targeting of YAP activity in vascular endothelial cells using F2RL1 peptide antagonist FSLLRY-NH2 inhibits fibrotic endothelial-to-mesenchymal transition and reduces UUO-induced kidney fibrosis (Table 1) [104]. Consistently, exosomes from human umbilical cord mesenchymal stem cells attenuated UUO-induced renal fibrosis by promoting ubiquitination or degradation of YAP [105].

Several studies have also described the role of YAP/TAZ during acute kidney injury (AKI)-induced fibrosis [32,106,107]. YAP is activated in the renal proximal tubule epithelial cells (RPTCs) after IRI as well in the kidney biopsy samples from patients with clinical AKI. The inhibition of YAP and TEAD interaction in RPTCs by verteporfin or conditional depletion of YAP in RPTCs using the mouse IRI model delayed renal function and recovery, indicating that YAP activation in RPTCs induces renal recovery from AKI. However, TAZ deletion showed no effect. EGFR-PI3K-Akt signaling-dependent activation of YAP was found to be critical for promoting renal recovery after IRI [32]. Similarly, Xu et al. described that increased YAP expression is associated with the repair of injured epithelia, although continuous YAP activation might be linked to the initiation of interstitial fibrosis and defective renal tubule differentiation during IR-induced AKI in rats [107]. This study suggested that YAP activation after renal ischemic injury could be beneficial or detrimental depending on the regulation of YAP activity. For example, a recent study demonstrated that a reprogramming factor KLF4 (Kruppel Like Factor 4)-mediated sustained activation of YAP in the post-acute phase of IR-induced AKI was related to declined renal function and augmented fibrosis. Activated YAP induced the expression of fibrogenic factors TGF-β and CTGF in renal tubular epithelial cells and stimulated the activation of interstitial fibroblasts [106]. Not only YAP, but Hippo kinases are also important regulators of renal fibrosis after AKI. An investigation demonstrated that conditional inactivation of the Hippo kinase *Sav1* in renal tubule cells using *Ksp^CreER^;Sav1^fl^*^/*fl*^ mice promoted renal interstitial fibrosis upon induction of AKI [36].

YAP activation was also found in hypertensive nephropathy, a CKD characterized by glomerular sclerosis, stiffness and renal fibrosis [103]. Zhang et al. showed that angiotensin (Ang) II-induced hypertensive renal injury promotes YAP activity, and causes increased proinflammatory and profibrotic factors including TNFα (tumor necrosis factor α), IL1β (interleukin 1β), MCP-1 (monocyte chemoattractant protein-1), TGF β, phospho-Smad3, and fibronectin. The blocking of YAP with verteporfin reversed AngII-induced renal inflammation and fibrosis [103]. The activation of YAP in RPTCs also drives renal interstitial fibrogenesis during diabetic neuropathy [33]. Similarly, the increased nuclear accumulation of TAZ was found in the tubulointerstitium of mice after renal injury caused by UUO or diabetic nephropathy correlating with fibrosis progression [31]. TAZ was also upregulated in human kidney (HK)-2 epithelial cells in a profibrotic environment induced by TGF-β1. The overexpression of TAZ promoted the activity of profibrotic factors, including CTGF and fibronectin, whereas the silencing of TAZ blocked the induction of profibrotic components [31]. Seo et al. [102] studied the role of the Hippo-Salvador pathway in the pathogenesis of renal tubulointerstitial fibrosis (TIF). Tubular epithelial cell (TEC)-specific genetic ablation of *Sav1* (Salvador homolog 1) exacerbated TIF and increased pathologic EMT (epithelial-mesenchymal transition)-like cells after UUO in mice. The authors showed that TEC-specific *Sav1* deletion increased TAZ, TGF-β, and β-catenin expression after UUO, which are required to promote EMT phenotypes. TAZ was also found to directly regulate the expression of TGF-β and TGF-β receptor II in vitro, indicating the essential role of the Hippo-Salvador pathway in TIF [102]. Another study reported MOB1-mediated role of the Hippo pathway in UUO-induced renal fibrosis [108]. This study showed that the focal adhesion molecule Kindlin-2 induces the MOB1 degradation by enhancing its interaction with E3 ligase praja2 and thus inhibiting LATS1 and YAP phosphorylation, which promotes nuclear translocation of YAP. Genetic depletion of Kindlin-2 led to the phosphorylation of Hippo/YAP signaling components MOB1, LATS1, and YAP in UUO mice and alleviated renal fibrosis. Similarly, siRNA against Kindlin-2 significantly attenuated UUO-induced renal fibrosis [108].

## 6. Hippo Signaling Pathway in Skin Fibrosis

Similar to its role in promoting fibrotic events in other organs, the Hippo pathway is known to contribute to the pathogenesis of skin fibrosis too [109,110]. The enhanced presence of YAP/TAZ was detected in skin biopsies from patients with systemic sclerosis (SSc), a complex fibrotic disease that may result from skin fibrosis and/or other organ fibrosis. Toyama et al. [110] showed a nuclear presence of YAP/TAZ within platelet-derived growth factor receptor beta (PDGFRβ)-positive fibroblasts in SSc biopsy sections. Furthermore, this study identified the potential use of dimethyl fumarate (DMF), which represents a class of molecules that enhance the intrinsic cellular antioxidant response (Table 1) [111], as an inhibitor of YAP/TAZ-mediated profibrotic response [110]. Indeed, DMF exposure reduced YAP/TAZ activity in response to matrix stiffness or TGFβ1-stimulation in SSc fibroblasts. Moreover, DMF-mediated inhibition of YAP/TAZ was effective in reducing skin fibrosis in a bleomycin-induced SSc mice model [110]. Mechanistically, in dermal fibroblasts, DMF treatment decreased the nuclear presence of TAZ/YAP by inhibiting the PI3K-AKT-GSK3-β pathway [110]. Similarly, elevated YAP1 expression was also found in Dupuytren disease—a condition characterized by the formation of myofibroblast-rich cords and nodules in the hands—where it partly co-localized in αSMA-positive cells [109]. Piersma et al. [109] demonstrated that YAP1-deficient myofibroblasts in Dupuytren disease showed a reduced expression of profibrotic genes such as *Acta2*, *Co1a1*, and *Ctgf*. The contractile ability of these myofibroblasts was also decreased due to YAP1 deficiency. Moreover, YAP1 is involved in the differentiation of dermal fibroblasts into highly contractile myofibroblasts in a TGFβ1-induced profibrotic environment. The siRNA-mediated knockdown of YAP1 in dermal fibroblasts abrogated the formation of α-SMA-induced stress fibers and interrupted collagen type I production [109]. YAP/TAZ knockdown also interrupted the functions of TGF-β1 and reduced its target gene expression by inhibiting Smad3 phosphorylation in dermal fibroblasts [112]. These findings suggest that targeting YAP/TAZ could be an effective therapeutic approach for treating fibrotic skin diseases.

## 7. Hippo Signaling Pathway in Fibrosis of Other Organs

YAP functions as an important regulator of TGF-β-induced profibrotic responses during retinal fibrosis in diabetic rats [113]. In cultured retinal Müller cells, YAP inhibition suppressed TGF-β1-stimulated myofibroblasts formation and ECM production, whereas YAP activation augmented TGF-β1-independent fibroblasts differentiation and ECM synthesis [113]. However, TGF-β1-mediated activation of YAP and the expression of fibrogenic factors are dependent on the PI3K/Akt signaling pathway [113]. Consistent with this, YAP/TAZ was reported to be crucial for TGF-β2-mediated conjunctival fibrosis [114]. The siRNA-mediated silencing of YAP significantly reduced α-SMA stress fibers and ECM components, such as fibronectin, and collagen type I and type IV through Smad2/3 signaling in cultured primary conjunctival fibroblasts exposed to TGF-β2 [114]. A study by Ou et al. [115] reported an increased expression of YAP/TAZ in stenotic intestines of Crohn’s disease patients, where they activated intestinal fibroblasts and thus promoted intestinal fibrosis. Mechanistically, the inhibition of ROCK1 (A Rho-associated coiled-coil-containing protein kinase 1) with Y27632 interrupted YAP/TAZ-dependent activation of fibrotic events in the intestinal fibroblasts and DSS-induced chronic colitis mice (Table 1) [115]. YAP also regulated the mesothelial to mesenchymal transition and peritoneal fibrosis during peritoneal dialysis [116].

**Table 1:** Targeting of the Hippo signaling pathway during fibrotic events.

Name of the Compounds	Mode of Actin	Experimental Study
Verteporfin [13,15,17,18,94,95,97]	Interference of YAP/TAZ-TEAD complex	▪ Prevents mouse HSCs activation and reduces fibrogenesis in mice exposed to CCl_4_ [13,94,95]. ▪ Aggravate IRI-induced liver fibrosis [97]. ▪ Reduces the kidney fibroblasts activation and UUO-induced kidney fibrosis in mice [15,17]. ▪ Inhibits the transition of cardiac fibroblasts into myofibroblasts [18].
XMU-MP-1 [39]	MST1/2 blocker	TAC-induced cardiac fibrosis in mice.
*Qishen* granules [43]	Disrupt YAP/TAZ expression	Reduce the fibrogenic protein expression in HF-hearts of the rat.
SKI [27]	Inducing proteasomal degradation of TAZ through the interaction with LIMD1	Inhibits the transition of primary rat cardiac fibroblast to myofibroblasts.
Lovastatin [44]	Suppression of YAP/TAZ signaling	Alleviates the AngII-induced cardiac fibrosis in mice.
Sulfur dioxide (SO2) [45]	Inhibits MST1/2	Prevent the myocardial fibrosis in diabetic rats
Melatonin [69]	interrupting the translocation of YAP into the nucleus	▪ Inhibits TGF-β1-induced myofibroblasts formation and ECM production by lung fibroblasts. ▪ Attenuates the interstitial lung fibrosis during bleomycin-induced IPF.
Icariin [70]	Inhibition of YAP function	Prevents the bleomycin-induced PF in rats.
Simvastatin [67]	Modulates YAP localization	Reduces established fibrosis in bleomycin-challenged mouse IPF.
PP242 [16]	Inhibits mTORC2 and suppresses YAP/TAZ	Inhibits the fibroblasts activation in UUO nephropathy induced mouse kidney fibrosis
FSLLRY-NH2 [104]	Selectively inhibits YAP activity	Blocks the endothelial-to-mesenchymal transition and reduces the UUO-induced kidney fibrosis.
*Kindlin-2* siRNA [108]	Induces the degradation of MOB1 and promotes the nuclear translocation of YAP	Attenuates UUO-induced renal fibrosis in mice.
Exosomes [105]	Promotes the βTrCP-mediated ubiquitination and degradation of YAP	Attenuates UUO-induced renal fibrosis.
Fluphenazine dihydrochloride, (a DRD2 antagonist) [100]	Suppress YAP activity and induce the type I interferon signaling	Reduces CCl4-induced liver fibrosis.
LPA [97]	YAP activator	Prevent IR-induced liver fibrogenesis in mice.
Dihydrexidine (a DRD1 agonist) [7]	Blocks YAP/TAZ	Reverse HSCs activation and bile duct ligation-induced hepatic fibrosis in mice
Tricyclic antidepressants [14]	Inhibit acid ceramidase and YAP/TAZ activity	Reduces human HSCs-activation
ω-3 PUFAs [98]	Promoting YAP/TAZ degradation in a proteasome-dependent manner	▪ Prevent the proliferation and activation of HSCs derived from humans and rats. ▪ Inhibits CCl_4_-induced liver fibrosis in mice.
*RhoA* siRNA [9]	Reduces YAP/TAZ expression and augments the LATS1/2 activity	Halt the liver fibrosis
Morin [99]	Reduces YAP/TAZ expression while activating the MST1 and LATS1 expression	▪ Blocks the HSC activation ▪ Alleviates liver fibrosis in diethylnitrosamine-induced rats.
Dimethyl fumarate (DMF) [110]	Inhibition of YAP/TAZ	▪ Inhibits stiffness or TGFβ1-induced activation of SSc fibroblasts. ▪ Reduce the skin fibrosis in bleomycin-induced SSc model mice.
Y27632 [115]	Inhibition of ROCK1	Inhibits YAP/TAZ-dependent activation of fibrotic events in intestinal fibroblasts and DSS-induced chronic colitis mice.

## 8. Conclusions and Remarks

The Hippo pathway has been established as a vital regulator of organ size control that controls cell proliferation, survival, and differentiation processes during organ development and regeneration. Here, we have summarized recent findings that provide evidence for the crucial role that Hippo pathway elements play in the pathophysiology of fibrotic disorders involving various cell types, especially fibroblasts. The Hippo signaling effector YAP/TAZ directly regulates the differentiation of fibroblasts into fibrogenic myofibroblasts, and blocking YAP/TAZ attenuates injury-induced fibroblast activation and organ fibrogenesis. The mechanotransduction function of YAP/TAZ is known to be crucial for the development of various organs including the lungs, kidneys, liver and skeletal muscle; however, the role of Hippo components in understanding the pathogenesis of muscle fibrosis remains less-studied. A better understanding of the classical and non-classical regulation of Hippo pathway components in a fibrotic environment is necessary to control the pathophysiology of fibrotic diseases. Likewise, a wide-ranging understanding of the cell-type and organ-specific functions of the Hippo pathway components at different stages of disease progression will enable the identification of suitable drug targets for the development of effective therapeutics against progressive fibrotic disorders.
